# The role of endotoxin/lipopolysaccharide in surgically induced tumour growth in a murine model of metastatic disease

**DOI:** 10.1038/sj.bjc.6694369

**Published:** 1999-12

**Authors:** G P Pidgeon, J H Harmey, E Kay, M Da Costa, H P Redmond, D J Bouchier-Hayes

**Affiliations:** Royal College of Surgeons in Ireland, Departments of Surgery andPathology, Beaumont Hospital, Beaumont, Dublin 9, Ireland

**Keywords:** endotoxin, lipopolysaccharide, metastasis, murine tumour, surgery

## Abstract

Surgical removal of a primary tumour is often followed by rapid growth of previously dormant metastases. Endotoxin or lipopolysaccharide, a cell wall constituent of Gram-negative bacteria, is ubiquitously present in air and may be introduced during surgery. BALB/c mice received a tail vein injection of 10^5^ 4T1 mouse mammary carcinoma cells. Two weeks later, animals were subjected to surgical trauma or an intraperitoneal injection of endotoxin (10 μg per animal). Five days later, animals which underwent open surgery, laparoscopy with air sufflation or received an endotoxin injection displayed increased lung metastasis compared to anaesthetic controls. These increases in metastatic tumour growth were reflected in increased tumour cell proliferation and decreased apoptosis within lung metastases. Circulating levels of the angiogenic cytokine, vascular endothelial growth factor (VEGF), were also elevated in these groups and correlated with increased plasma levels of endotoxin. Endotoxin treatment for 18 h (>10 ng ml^–1^) directly up-regulated VEGF production by the 4T1 tumour cells in vitro. Metastatic tumour growth in mice undergoing carbon dioxide laparoscopy, where air is excluded, was similar to anaesthetic controls. These data indicate that endotoxin introduced during surgery is associated with the enhanced growth of metastases following surgical trauma, by altering the critical balances governing cellular growth and angiogenesis. © 1999 Cancer Research Campaign


*British Journal of Cancer* (1999) **81**(8), 1311–1317
© 1999 Cancer Research Campaign
Article no. bjoc.1999.0846

### The role of endotoxin/lipopolysaccharide in surgically induced tumour growth in a murine model of metastatic disease

**GP Pidgeon[1], JH Harmey[1], E Kay[2], M Da Costa[1], HP Redmond[1] and DJ Bouchier-Hayes[1]**

Royal College of Surgeons in Ireland, Departments of [1]Surgery and [2]Pathology, Beaumont Hospital, Beaumont, Dublin 9, Ireland

**Summary** Surgical removal of a primary tumour is often followed by rapid growth of previously dormant metastases. Endotoxin or lipopolysaccharide, a cell wall constituent of Gram-negative bacteria, is ubiquitously present in air and may be introduced during surgery. BALB/c mice received a tail vein injection of $10^5$ 4T1 mouse mammary carcinoma cells. Two weeks later, animals were subjected to surgical trauma or an intraperitoneal injection of endotoxin (10 μg per animal). Five days later, animals which underwent open surgery, laparoscopy with air sufflation or received an endotoxin injection displayed increased lung metastasis compared to anaesthetic controls. These increases in metastatic tumour growth were reflected in increased tumour cell proliferation and decreased apoptosis within lung metastases. Circulating levels of the angiogenic cytokine, vascular endothelial growth factor (VEGF), were also elevated in these groups and correlated with increased plasma levels of endotoxin. Endotoxin treatment for 18 h (>10 ng $ml^{-1}$) directly up-regulated VEGF production by the 4T1 tumour cells in vitro. Metastatic tumour growth in mice undergoing carbon dioxide laparoscopy, where air is excluded, was similar to anaesthetic controls. These data indicate that endotoxin introduced during surgery is associated with the enhanced growth of metastases following surgical trauma, by altering the critical balances governing cellular growth and angiogenesis. © 1999 Cancer Research Campaign

**Keywords:** endotoxin; lipopolysaccharide; metastasis; murine tumour; surgery

Rapid growth of previously dormant metastases following surgical removal of a primary tumour is well documented (Arai et al, 1992; Kodama et al, 1992). Up to 50% of patients diagnosed with primary cancer already have metastatic deposits (Fidler and Ellis, 1994). The mechanisms controlling growth and dormancy of metastases is an area of intense investigation. Primary tumours have been shown to inhibit the growth of their own metastases by the secretion of angiogenesis inhibitors such as angiostatin and endostatin (O'Reilly et al, 1994; O'Reilly et al, 1997). Tumour manipulation during surgery may increase tumour cell dissemination into the bloodstream resulting in the seeding of tumour cells in distant organs and the establishment of metastases (Hansen et al, 1995). Surgical removal of a primary tumour may therefore not only facilitate the seeding of metastases, but also create a permissive environment for their subsequent growth by removing the source of angiogenesis inhibitors. We have previously reported that the growth of metastases following excision of a B16 melanoma primary tumour was increased when a simultaneous laparotomy or laparoscopy was performed at the time of excision (Da Costa et al, 1998). The effect of the surgical insult itself on the growth of metastases therefore merits separate investigation.

Tumour cell metastasis is a complex multistep process involving a number of cell types, cytokines and pathways (Crissman et al, 1988; Naylor and Balkwill, 1995). Angiogenesis, the formation of new blood vessels, is essential for the growth of primary tumours and established metastases (Folkman, 1990; Fidler and Ellis, 1994). In the absence of neovascularization, solid tumours are restricted to a diameter of 2–3 $mm^3$ (Folkman, 1990). New vessel formation is associated with increased metastasis as the new vessels allow tumour cells access to the circulation (Weidner et al, 1991; Anan et al, 1996). Net angiogenesis is a consequence of the balance between pro- and anti-angiogenic molecules. Vascular endothelial growth factor (VEGF), also known as vascular permeability factor (VPF), is a potent angiogenic cytokine stimulating the growth and differentiation of endothelial cells (Peters et al, 1993). It also increases vascular permeability resulting in leaky blood vessels thereby facilitating tumour cell extravasation, a critical step in the metastatic cascade (Clauss et al, 1990; Marmé, 1996). VEGF is produced by a variety of host inflammatory and tumour cells (Sunderkotter et al, 1994; Freeman et al, 1995; Harmey et al, 1998).

In addition to new vessel formation, net tumour growth depends on the balance between tumour cell proliferation and apoptosis (Holmgren et al, 1995). Apoptosis, or programmed cell death, is characterized by single-cell death in the midst of living cells. When proliferation equals the rate of apoptosis, no net growth results and the tumours enter a state of quiescence or dormancy.

Endotoxin or lipopolysaccharide (LPS) is a cell wall constituent of Gram-negative bacteria that is present ubiquitously in the atmosphere at concentrations of approximately 1 μg $m^{-3}$ (Rylander et al, 1989). Another major source of endotoxin is endogenous gut bacteria. Abdominal contamination with airborne factors during surgery has previously been shown to cause immunological alterations and bacterial translocation (Watson et al, 1995). Recent reports have shown that endotoxin is angiogenic, inducing neovascularization in both the mesenteric window and corneal implant model (Li et al, 1991; Mattsby-Baltzer et al, 1994; Kenyon et al, 1996).

*Received 22 February 1999*
*Revised 27 May 1999*
*Accepted 3 June 1999*

*Correspondence to:* JH Harmey

We hypothesized that open surgery (laparotomy) results in increased metastatic tumour growth and that this increase is due, at least in part, to LPS introduced during the surgical procedure and/or LPS translocation within the peritoneal cavity. To investigate the role of surgery and endotoxin in metastatic growth we used a murine model of metastatic disease where no primary tumour is established. In this model, alterations in tumour growth cannot be attributed to the removal of angiogenesis inhibitors, such as angiostatin, derived from primary tumours. We assessed the effects of laparotomy, air laparoscopy, laparoscopy with air exclusion under carbon dioxide ($CO_2$) and endotoxin injection (10 µg per animal) on metastatic tumour burden. Increased metastatic burden, elevated serum VEGF, elevated circulating LPS, increased tumour cell proliferation and decreased apoptosis were identified in animals subjected to laparotomy or air laparoscopy. In the $CO_2$ laparoscopy group, these parameters were similar to controls. In animals receiving endotoxin injection, metastatic growth was similar to that observed in the laparotomy group.

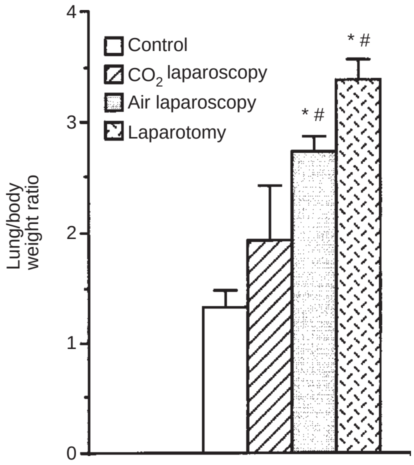

**Figure 1** Metastatic burden in mice following surgery. Surgery was performed 2 weeks after tail vein injection of $10^5$ 4T1 mammary cells. Five days later animals were sacrificed and lungs were removed. Tumour burden is expressed as lung weight/body weight × 100 (mean ± s.d., $n = 8$ per group). A significant increase in metastatic growth was observed following laparotomy and air laparoscopy *($P < 0.0001$) compared to controls or $CO_2$ laparoscopy #($P < 0.001$)

### MATERIALS AND METHODS

### Animals

Male 6- to 8-week old BALB/c mice (Charles River Institute, Margate, Kent, UK) were used. The animals were acclimatized for 1 week and caged in groups of five or less in an air-conditioned room at ambient temperature of 21–22°C and 50% relative humidity under a 12-h light–dark cycle (lights at 08.00). Animals were housed in a licensed biomedical facility (RCSI Department of Surgery, Beaumont Hospital) and all procedures were carried out under animal license guidelines of the Ministry of Health, Ireland. Animals had ad libitum access to animal chow (WM Connolly & Sons Ltd, Kilkenny, Ireland) and water.

### Cell culture and injection of cells

The spontaneously metastasizing mammary adenocarcinoma cell line, 4T1, was generously provided by Mr E Coveny (Waterford Regional Hospital, County Waterford, Ireland). All cell culture reagents were obtained from Gibco-BRL (Paisley, UK). 4T1 cells were maintained in RPMI-1640 supplemented with 10% heat-inactivated fetal calf serum (FCS), 100 U $ml^{-1}$ penicillin and 100 µg $ml^{-1}$ streptomycin sulphate in a humidified atmosphere of 5% $CO_2$ in air at 37°C. Cells at 80% confluency were removed by trypsinization and washed three times in $Ca^{++}$/$Mg^{++}$-free phosphate-buffered saline (PBS). Cells were resuspended at $5 \times 10^5$ $ml^{-1}$ in PBS and 200 µl injected into each mouse via the lateral tail vein following intramuscular administration of the anaesthetic and vasodilator hypnorm (Janssen, Buckinghamshire, UK).

### Experimental design – surgical groups

Two weeks after tumour cell injection, mice were randomized into four groups ($n$ = 8 per group). A control group received anaesthesia (Halothane, Rhone-Poulenc Rorer Ltd, Dublin, Ireland) for 30 min, a laparoscopy group received air sufflation of the peritoneal cavity for a period of 30 min, a group received laparoscopy with $CO_2$ instead of air (3 mmHg constant pressure) and a laparotomy group underwent a midline incision along the peritoneum with periodical agitation of the intestines over a 30-min time interval.

Five days later, the animals were weighed, blood was collected and animals sacrificed by cervical dislocation. Lungs were excised and weighed immediately. The percentage lung weight: body weight (lung weight/body weight × 100) was used as an indicator of metastatic burden, similar to the method used previously to assess kidney hypertrophy (Nakamura et al, 1996). The lungs were fixed in 10% formalin and processed for histology.

### Experimental design – injection groups

In a second set of experiments, animals received tumour cells via the lateral tail vein as before. Two weeks later the animals were divided into two groups ($n$ = 8 per group). The control group received a 200 µl intraperitoneal (i.p.) injection of sterile saline and the experimental group received a 200 µl injection of 50 µg $ml^{-1}$ commercial LPS (Sigma Corp., Dublin, Ireland) in saline, i.e. 10 µg LPS per mouse. Parameters were measured as for the surgical groups.

### Serum collection

Mice were anaesthetized with halothane and their chests cleaned with ethanol. Blood was obtained via closed cardiac puncture by means of a 22-gauge hypodermic needle and a subxiphoid approach. Blood was allowed to clot for 2 h at room temperature and centrifuged for 20 min at 1100 ***g***. Serum was removed, filtered through a 0.22-µm filter and stored at –80°C. VEGF was measured by enzyme-linked immunosorbent assay (ELISA) according to manufacturer's instructions (R&D Systems, Oxford, UK).

### Histology

Paraffin-embedded sections were stained with haematoxylin and eosin and a mitotic index (MI) estimated using a 1 $mm^3$ grid

 *© 1999 Cancer Research Campaign*

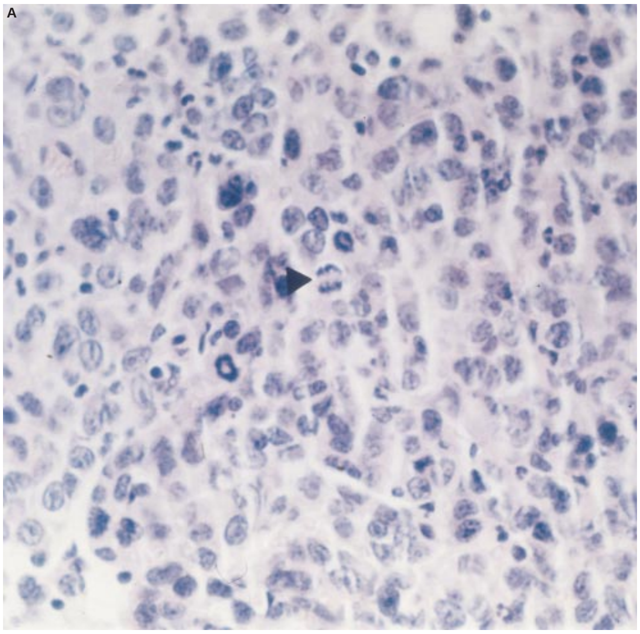

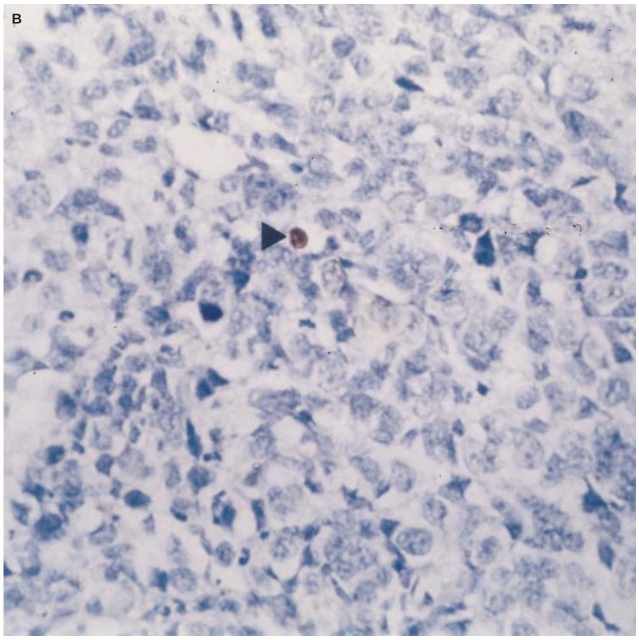

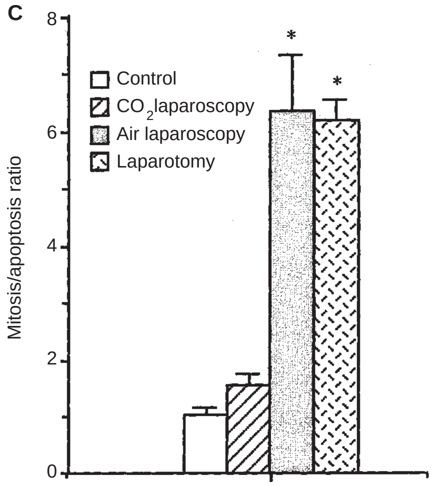

**Figure 2** Mitosis and apoptosis within lung metastases following surgery. **(A)** Mitotic cells in lung metastases. Arrow indicates cell during anaphase with chromosome separation to two separate poles (magnification × 400). **(B)** Apoptotic cells in lung metastases. Arrow indicates apoptotic cell following TUNEL staining as described in Materials and Methods (magnification × 400). **(C)** Mitosis: apoptosis ratios within metastases following surgery. The number of mitotic and apoptotic cells (mean of ten fields) were estimated for each group and the ratio calculated. Data represent mean ± s.d. ($n$ = 8 per group). A significant increase in mitosis:apoptosis ratios were observed following laparotomy and air laparoscopy *($P < 0.01$) compared to controls or $CO_2$ laparoscopy. There was no significant difference between the controls and $CO_2$ laparoscopy groups ($P$ = NS)

counting an average of 500 tumour cells mm$^{-3}$. Ten fields were scored by two independent observers (GP and JH) in a blinded fashion. Mitotic cells were identified morphologically and the mean number of mitotic cells in ten fields used as the mitotic index.

Apoptotic cells were stained using the in situ cell death detection kit according to manufacturer's instructions (Boehringer Mannheim, East Sussex, UK). Peroxidase activity was visualized by the precipitation of 3,3´-diaminobenzidene. Apoptotic cells stained brown and were counted as above. The apoptotic index (AI) was calculated as the mean number of positively stained cells in ten fields.

### VEGF production by 4T1 cells in vitro

4T1 tumour cells were plated at $2 \times 10^4$ cells well$^{-1}$ in 96-well plates. Sixteen hours later LPS (0–10 µg ml$^{-1}$) was added. Eighteen hours later supernatants were collected and stored at –80°C for analysis. Cells were washed twice with PBS and total protein measured using the Bicinchoninic Acid method (Pierce, IL, USA). Supernatants were assayed for VEGF by ELISA (R&D Systems, Oxford, UK) and results expressed as VEGF pg µg$^{-1}$ total protein.

### Plasma LPS measurement

Animals ($n$ = 5) were inoculated with 4T1 tumour cells as before, and randomly divided into surgical and injection groups as previously described in the experimental design section. Four hours after surgical treatment or injection, blood was harvested via closed cardiac puncture into pyrogen-free Coatex Endo tubes (Chromogenix AB, Mölndak, Sweden) and plasma separated by centrifugation at 2200 ***g*** for 15 min. Plasma LPS levels were measured using the Limulus Amebocyte Lysate assay (Chromogenix AB, Mölndak, Sweden) according to manufacturer's protocol and results expressed as LPS EU ml$^{-1}$.

### Statistical analysis

Statistical comparison between surgical groups was carried out with analysis of variance (ANOVA) and Scheffe post-hoc correction using DataDesk 4.1™ (Data Description Inc., Ithaca, NY, US). The two injection groups were analysed using a two-sample $t$-test in DataDesk 4.1™. Results are expressed as means ± standard deviation (s.d.). Data were taken as significant where $P < 0.05$. A

*© 1999 Cancer Research Campaign*

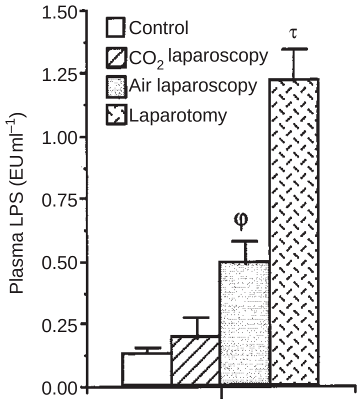

**Figure 3** Plasma LPS levels following surgery. Blood was collected by cardiac puncture and LPS levels were examined 4 h post-surgery by the LAL assay as described in Materials and Methods. Data represent mean ± s.d. (*n* = 8 per group). Laparotomy τ ($P < 0.0001$) and air laparoscopy φ ($P < 0.03$) both resulted in significantly elevated levels of circulating LPS compared to the controls receiving anesthesia alone. No significant difference was observed between the control and $CO_2$ laparoscopy groups ($P$ = NS)

Pearson Product Moment correlation was calculated for serum VEGF levels in relation to plasma LPS concentrations.

### RESULTS

### Effect of surgery on metastatic tumour growth

4T1 mammary adenocarcinoma cells spontaneously metastasize to the lungs. A preliminary study established that lung metastases were visibly present 14 days after tail vein injection of $10^5$ 4T1 tumour cells. No metastases were apparent in other organs at this time or at the time of sacrifice (5 days later). There was no significant difference in body weights between groups, indicating that the animals were not cachectic ($P$ = not significant (NS)) and no apparent oedema was observed. Both laparotomy (3.37 ± 0.14) and air laparoscopy (2.73 ± 0.11) groups had a significantly ($P < 0.0001$) higher metastatic burden than controls (1.32 ± 0.12), indicated by lung/body weight measurements. A metastatic burden similar to controls was observed in animals undergoing $CO_2$ laparoscopy, where air is excluded (1.94 ± 0.42, $P$ = NS) (Figure 1). Surface examination of the lungs in the laparotomy and air laparoscopy groups revealed multiple macroscopic tumour metastases in comparison with the other two groups.

We assessed tumour cell mitosis and apoptosis within lung metastases. Similar trends were observed to those seen for the metastatic burden. Representative sections with highlighted mitotic and apoptotic cells are shown in Figure 2A and 2B, respectively. The laparotomy (8.33 ± 0.21) and air laparoscopy (7.99 ± 0.09) groups had significantly ($P < 0.0001$) higher mitotic indices compared with control (4.39 ± 0.63) or $CO_2$ laparoscopy (4.71 ± 0.15) groups. Laparotomy (1.31 ± 0.13) and laparoscopy with air (1.33 ± 0.42) resulted in significantly ($P < 0.01$) lower apoptotic indices compared with controls (4.46 ± 1.13). In contrast, laparoscopy with $CO_2$ resulted in a similar mitotic (4.39 ± 0.63) and apoptotic index to controls (3.17 ± 0.76, $P$ = NS).

As net tumour growth is ultimately determined by the relative numbers of proliferating and apoptotic tumour cells, the ratio of MI/AI was calculated as described. Laparotomy (6.20 ± 0.35) and laparoscopy with air (6.35 ± 1.00) resulted in significantly ($P < 0.01$) higher ratios compared with either $CO_2$ laparoscopy (1.54 ± 0.19) or control (1.02 ± 0.15) groups (Figure 2C).

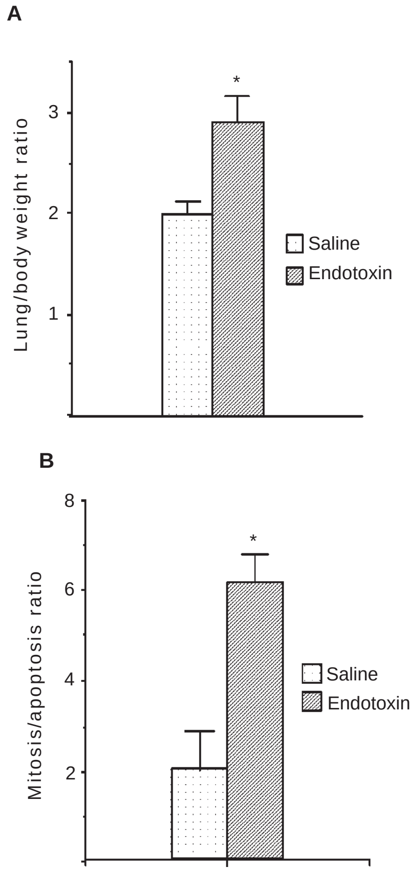

**Figure 4** **(A)** Lung:body weight in mice following saline or LPS injection. Two weeks after tail vein injection of $10^5$ 4T1 mammary cells animals received an injection of saline or 10 μg LPS. Five days later animals were sacrificed and lungs were removed. Data represent mean ± s.d. (*n* = 5 per group). A significantly higher tumour burden (indicated by lung/body weights) was observed following LPS injection *($P < 0.0002$) compared to animals receiving an equivalent injection of saline. (**B**) Mitosis:apoptosis ratios within lung metastases of mice following saline or LPS injection. Data represent mean ± s.d. (*n* = 5 per group). A significantly higher * ($P < 0.02$) ratio was observed following LPS injection compared to animals receiving saline injection

### Plasma LPS levels following surgery

Laparotomy (1.22 ± 0.18 EU ml$^{-1}$) and air laparoscopy (0.50 ± 0.15 EU ml$^{-1}$) groups had significantly ($P < 0.05$) higher levels of circulating LPS than control (0.13 ± 0.09 EU ml$^{-1}$). Animals that received laparoscopy with air excluded ($CO_2$ laparoscopy) had levels of LPS comparable with controls (0.19 ± 0.14 EU ml$^{-1}$, $P$ = NS versus control) (Figure 3).

### Effect of LPS injection on metastatic tumour growth

Endotoxin injection resulted in a significantly ($P < 0.001$) higher metastatic burden compared with the group receiving the saline injection (2.84 ± 0.20 vs 1.99 ± 0.10 respectively) (Figure 4A). Endotoxin injection resulted in a similar metastatic burden to that observed (Figure 1) in the laparotomy and air laparoscopy groups (3.37 ± 0.14 and 2.73 ± 0.11 respectively). This higher metastatic

 © 1999 Cancer Research Campaign

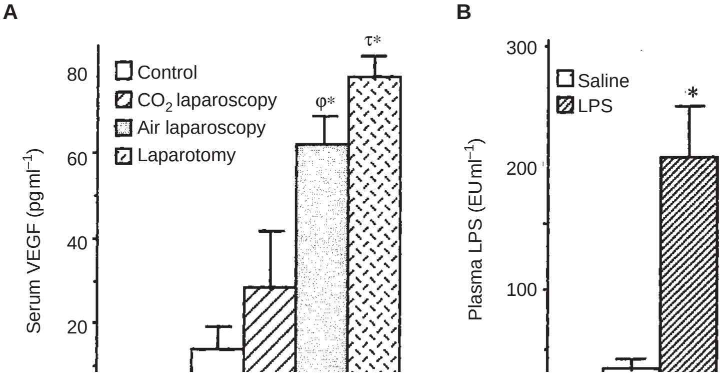

**Figure 5** **(A)** Serum VEGF in mice following surgery. Five days post-operatively blood was obtained by cardiac puncture and VEGF was assessed by quantitative ELISA. Data represent mean ± s.e.m. ($n$ = 8 per group). A significant increase in circulating levels of VEGF was observed following laparotomy and laparoscopy with air compared to controls * ($P$ < 0.0001) or $CO_2$ laparoscopy groups, φ ($P$ < 0.01) τ ($P$ < 0.001). (**B**) Serum VEGF levels following i.p. injection of saline or 10 µg LPS. Data represent mean ± s.d. ($n$ = 5 per group). LPS injection resulted in a significant* ($P$ < 0.0003) increase in circulating levels of VEGF compared to animals receiving a saline injection

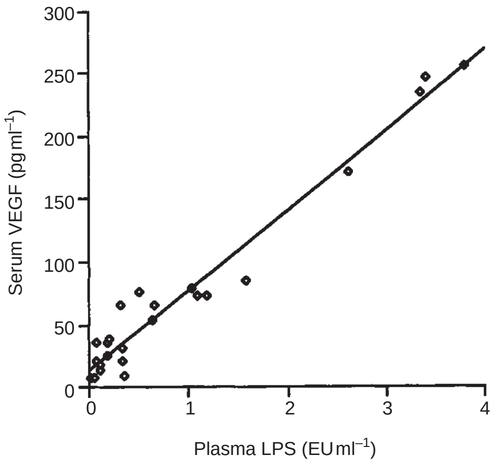

**Figure 6** Circulating VEGF in relation to plasma LPS levels ($n$ = 24). A strong positive correlation ($r$ = 0.966) was observed between serum VEGF and plasma LPS levels

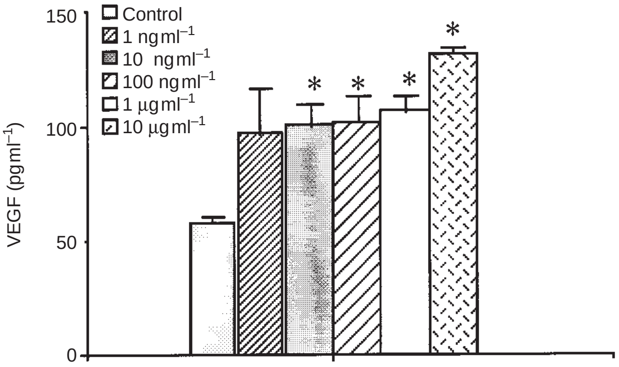

**Figure 7** VEGF production by 4T1 tumour cells following stimulation with LPS. 2 × $10^4$ 4T1 cells were plated out in 96-well plates, and 18 h later they were incubated with different concentrations of LPS. LPS at concentrations above 10 ng ml$^{-1}$ resulted in significantly * ($P$ < 0.05) higher levels of VEGF (pg µg$^{-1}$ total cell protein). Data represent mean ± s.d. of three experiments carried out in duplicate burden was accompanied by a higher mitotic index in the endotoxin injection group (7.68 ± 0.42) compared with the saline injection group (4.74 ± 1.71). In addition, a lower apoptotic index was observed in the endotoxin group (1.27 ± 0.19) compared with the saline group (2.98 ± 0.91). The MI/AI ratio in the endotoxin injection group (6.18 ± 0.64) was significantly ($P$ < 0.02) higher than the ratio observed following saline injection (2.02 ± 0.88) (Figure 4B) and similar to the MI/AI ratio in the surgical groups exposed to air – laparotomy (6.20 ± 0.35) and laparoscopy with air sufflation (6.35 ± 1.00).

### Serum VEGF levels following surgery or injection

Laparotomy (77.92 ± 5.00 pg ml$^{-1}$) and air laparoscopy (61.97 ± 7.14 pg ml$^{-1}$) resulted in significantly ($P$ < 0.0001) elevated post-operative levels of circulating VEGF compared with either control (14.28 ± 5.44 pg ml$^{-1}$) or $CO_2$ laparoscopy groups (28.41 ± 13.70 pg ml$^{-1}$) (Figure 5A). No significant difference was observed between the control and $CO_2$ laparoscopy group ($P$ = NS). The presence of high levels of this pro-angiogenic factor in the circulation following surgery implicates an angiogenic mechanism in the enhanced tumour growth observed in the surgical groups exposed to air and in animals receiving endotoxin injection (207.85 ± 42.74 pg ml$^{-1}$).

Endotoxin injection resulted in significantly ($P$ < 0.003) higher levels of serum VEGF than saline injection (33.88 ± 7.34 pg ml$^{-1}$) (Figure 5B). This level is almost threefold that observed in animals undergoing laparotomy. This may be due to the high levels of circulating LPS following i.p. injection of 10µg LPS (3.13 ± 0.14 EU ml$^{-1}$), which was also threefold higher than plasma LPS in the laparotomy group (1.22 ± 0.18 EU ml$^{-1}$). A strong positive significant correlation ($r$ = 0.966) was observed between circulating VEGF and LPS levels, when all groups were examined in parallel (Figure 6). Therefore we investigated the effect of LPS on VEGF production by 4T1 tumour cells in vitro.

### Tumour cell production of VEGF following LPS stimulation

LPS treatment (10 ng, 100 ng, 1 µg and 10 µg) directly up-regulated VEGF production by 4T1 tumour cells in vitro (93.27 ± 18.14, 95.94 ± 19.83, 100.30 ± 16.07 and 117.22 ± 23.78

*© 1999 Cancer Research Campaign*

pg $mg^{-1}$ protein respectively, $P < 0.02$ vs untreated control cells $59.10 \pm 3.73$ pg $mg^{-1}$ protein) (Figure 7).

### DISCUSSION

We identified increased metastatic tumour growth in mice which underwent laparotomy or air laparoscopy, where the peritoneum was insufflated with air, compared to controls which received anaesthetic only. Mice that received a $CO_2$ laparoscopy, where air is excluded, had a metastatic burden comparable to the control group, implicating an airborne factor in this increased metastatic growth. The increases in metastatic burden coincided with significantly higher tumour cell mitosis and lower apoptosis within lung metastases of both laparotomy and air laparoscopy groups. The removal of a primary tumour (and consequently the anti-angiogenic factors it produces) has been implicated in post-operative tumour recurrence (O’Reilly et al, 1994, 1997). In our model there is no primary tumour established. Therefore, the increased metastatic growth observed in animals exposed to air during the surgical procedure cannot be attributed to the removal of anti-angiogenic factors. The tumour enhancing effect of open surgery has been demonstrated previously (Skipper et al, 1989; Arai et al, 1992; Kodama et al, 1992), but the mechanisms by which surgery increases tumour growth have remained unclear.

Both laparotomy and air contamination of the peritoneum have previously been shown to result in bacterial translocation across the gut (Watson et al, 1995), and we observed high plasma levels of LPS in animals 4 h after i.p injection of LPS. Bacterial translocation results in the release of LPS from endogenous gut bacteria, potentiating the inflammatory response. Our study suggests that air contamination during the surgical procedure is one of the stimuli responsible for the enhanced tumour growth and angiogenesis observed following open surgery. In animals which received endotoxin injection, metastatic burden was increased compared to those receiving a saline injection. Again, this increase was reflected in higher tumour cell proliferation and decreased apoptosis within the lung metastases. LPS has been implicated in cellular proliferation and differentiation, possibly through the phosphorylation and activation of several protein kinases (Weinstein et al, 1992; Shapira et al, 1994).

VEGF is the most potent angiogenic factor known (Peters et al, 1993). VEGF production can be regulated by a range of effector molecules and cytokines. Open surgery and air laparoscopy resulted in significantly elevated levels of circulating serum VEGF compared with either the $CO_2$ laparoscopy group or controls. Animals receiving an endotoxin injection also had elevated serum VEGF indicating that, in addition to promoting tumour growth, LPS induces the release of the pro-angiogenic cytokine VEGF. When the levels of plasma LPS were compared with circulating VEGF in all groups, a strong positive correlation was observed and we demonstrated that LPS directly increased VEGF production by tumour cells in vitro.

Our results indicate that endotoxin, introduced into the circulation during the surgical procedure, augments metastatic growth by increasing tumour cell proliferation, decreasing tumour cell apoptosis and increasing production of the angiogenic factor VEGF. It is therefore likely that endotoxin plays a role in tumour recurrence following surgical trauma. Thus, increased metastatic tumour growth following removal of a primary tumour may not be entirely due to the removal of angiogenesis inhibitors produced by the primary tumour. Our findings may be of particular relevance to patients with a previous history of cancer undergoing surgery for an unrelated complaint. We are currently investigating whether the administration of blocking antibodies to LPS in the peri-operative period attenuates surgically induced increases in metastatic tumour growth.

### ACKNOWLEDGEMENTS

This work was supported by grants from the Royal College of Surgeons in Ireland and the Charitable Infirmary Charitable Trust. We are grateful to Quaing Di Wu, Claire Condron, Patricia Tibett and the staff of the biomedical facility (Beaumont Hospital) for their excellent technical assistance.

### REFERENCES

Anan K, Morisaki T, Katano M, Ikubo A, Kitsuki H, Uchiyama A, Kuroki S, Tanaka M and Torisu M (1996) Vascular endothelial growth factor and platelet-derived growth factor are potential angiogenic and metastatic factors in human breast cancer. *Surgery* **119**: 333–339

Arai MD, Asakura T and Nemir P (1992) Effect of local tumour removal and retained oncolysate on lung metastasis. *Ann Surg Res* **53**: 30–38

Clauss M, Gerlach M, Gerlach H, Brett J, Wang F, Familletti PC, Pan Y-CE, Olander JV, Connolly DT and Stern D (1990) Vascular Permeability Factor: a tumour-derived polypeptide that induces endothelial cell and monocyte procoagulant activity, and promotes monocyte migration. *J Exp Med* **172**: 1535–1545

Crissman JD, Hatfield J, Menter D, Sloane B and Honn K (1988) Morphological study of the interaction of intravascular tumour cells with endothelial cells and subendothelial matrix. *Cancer Res* **48**: 4065–4072

Da Costa ML, Redmond HP and Bouchier-Hayes D (1998) The effect of laparotomy and laparoscopy on the establishment of spontaneous tumor metastases. *Surgery* **124**: 516–525

Fidler IJ and Ellis LM (1994) The implications of angiogenesis for the biology and therapy of cancer metastases. *Cell* **79**: 185–188.

Folkman J (1990) What is the evidence that tumours are angiogenesis dependent. *J Natl Cancer Inst* **82**: 4–6

Freeman MR, Schneck FX, Gagnon ML, Corless C, Soker S, Niknejad K, Peoples GE and Klagsbrun M (1995). Peripheral blood T lymphocytes and lymphocytes infiltrating human cancers express vascular endothelial growth factor: a potential role for T cells in angiogenesis. *Cancer Res* **55**: 4140–4145.

Hansen E, Norbert W, Knuechel R and Ruschoff J (1995) Tumour cells in blood shed from the surgical field. *Arch Surg* **130**: 387–393.

Harmey JH, Dimitriadis E, Kay E, Redmond HP and Bouchier-Hayes DJ (1998) Regulation of macrophage production of vascular endothelial growth factor (VEGF) by hypoxia and transforming growth factor β-1. *Ann Surg Oncol* **5**: 271–278.

Holmgren L, O’Reilly MS and Folkman J (1995) Dormancy of micrometastases: balanced proliferation and apoptosis in the presence of angiogenesis suppression. *Nat Med* **1**: 149–153.

Jiang WG, Puntis MCA and Hallett MB (1994) Molecular and cellular basis of cancer invasion and metastasis: implications for treatment. *Br J Surg* **81**: 1576–1590.

Kenyon BM, Voest EE, Chen CC, Flynn E, Folkman J and D’Amato RJ (1996) A model of angiogenesis in the mouse cornea. *Invest Ophth Vis Sci* **37**: 1625–1632.

Kodama M, Kodama T, Nishi Y and Totani R (1992) Does surgical stress cause tumour metastasis? *Anticancer Res* **12**: 1603–1616.

Li WW, Grayson G, Folkman J and D’Amore PA (1991) Sustained-release endotoxin. A model for inducing corneal neovascularisation. *Invest Ophth Vis Sci* **32**: 2906–2911.

Marmé D (1996) Tumour angiogenesis: the pivotal role of vascular endothelial growth factor. *World J Urol* **14**: 166–174.

Mattsby-Baltzer I, Jakobsson A, Sörbo J and Norrby K (1994) Endotoxin is angiogenic. *Int J Exp Pathol* **75**: 191–196.

Nakamura T, Ebihara I, Fukai M, Tomino Y and Koide H (1996) Modulation of endothelin family gene expression in renal hypertrophy. *Nephron* **73**: 228–234.

Naylor MS and Balkwill FR (1995) The role of cytokines in tumour progression. In: *Cell Proliferation and Cancer, Regulatory Mechanisms of Neoplastic Cell Growth*, Pusztai L, Lewis CE and Yap E (eds.), pp. 105–130. Oxford University Press: New York

 *© 1999 Cancer Research Campaign*

O'Reilly MS, Holmgren L, Shing Y, Chen C, Rosenthal RA, Moses M, Lane WS, Cao Y, Sage EH and Folkman J (1994) Angiostatin: a novel angiogenesis inhibitor that mediates the suppression of metastases by a Lewis lung carcinoma. *Cell* **79**: 315–328.
O'Reilly MS, Boehm T, Shing Y, Fukai N, Vasios G, Lane WS, Flynn E, Birkhead JR, Olsen BR and Folkman J (1997) Endostatin: An endogenous inhibitor of angiogenesis and tumour growth. *Cell* **88**: 277–285.
Peters KG, DeVries C and Williams LT (1993) Vascular endothelial growth factor expression during embryogenesis and tissue repair suggests a role in endothelial differentiation and blood vessel growth. *Proc Natl Acad Sci (USA)* **90**: 8915–8919.
Rylander R, Bake B, Fischer J and Helander I (1989) Pulmonary function and symptoms after inhalation of endotoxin. *Am Rev Respir Dis* **140**: 981–986.
Shapira L, Takashiba S, Champagne C, Amar S and VanDyke T (1994) Involvement of protein kinase C and protein tyrosine kinase in lipopolysaccharide-induced TNF-$\alpha$ and IL-1$\beta$ production by human monocytes. *J Immunol* **153**: 1818–1824.
Skipper D, Jeffrey MJ, Cooper AJ, Alexander P and Taylor I (1989) Enhanced growth of tumour cells in healing colonic anastomoses and laparotomy wounds. *Int J Colorect Dis* **4**: 172–177
Sunderkotter C, Steinbrink K, Goebeler M, Bhardwaj R and Sorg C (1994) Macrophages and angiogenesis. *J Leukoc Biol* **55**: 410–422.
Watson RWG, Redmond HP, McCarthy J, Burke PE and Bouchier-Hayes D (1995) Exposure of the peritoneal cavity to air regulates early inflammatory responses to surgery in a murine model. *Br J Surg* **82**: 1060–1065
Weidner MD, Semple JP, Welch WR and Folkman J (1991) Tumour angiogenesis and metastasis – correlation in invasive breast carcinoma. *N Engl J Med* **324**: 1–8.
Weinstein SL, Sanghera J, Lemke K, Defranco A and Pelech S (1992) Bacterial lipopolysaccharide induces tyrosine phosphorylation and activation of mitogen-activated protein kinases in macrophages. *J Biol Chem* **267**: 14955–14962.

© 1999 Cancer Research Campaign
